# Use of Foliar Biostimulants in Durum Wheat: Understanding Its Potential in Improving Agronomic and Quality Responses Under Mediterranean Field Conditions

**DOI:** 10.3390/plants14152276

**Published:** 2025-07-24

**Authors:** Angelo Rossini, Roberto Ruggeri, Francesco Rossini

**Affiliations:** Department of Agriculture and Forest Sciences, University of Tuscia, Via S. Camillo de Lellis, 01100 Viterbo, Italy; angelo.rossini@unitus.it (A.R.); rossini@unitus.it (F.R.)

**Keywords:** biostimulants, *Codium fragile*, durum wheat, vaterite

## Abstract

Foliar application of biostimulants can be a valid option to reach the goal of sustainable intensification in agriculture, especially in extensive crops such as durum wheat. However, due to the wide range of active ingredients and their mixtures available in the market, the need to select the most efficient product in a specific growing environment is of dramatic importance to achieve remarkable results in yield and grain quality. To analyze the potential of different active ingredients, a field trial was performed in two consecutive growing seasons (2023 and 2024) under Mediterranean climatic conditions. A randomized block design with three replicates was used. Durum wheat cultivar “Iride” was treated with the following five foliar biostimulants in comparison with the untreated control (T0): seaweed and plant extracts (T1); micronized vaterite (T2); culture broth of *Pseudomonas protegens* (T3); humic and fulvic acids (T4); organic nitrogen fertilizer (N 5%) containing glycine betaine (T5). Biostimulant treatment was applied at the end of tillering and at heading. Root length, chlorophyll content, grain yield, yield components and grain quality were measured and subjected to a one-way analysis of variance. As compared to the control, seaweed and plant extracts as well as micronized vaterite showed the best results in terms of grain yield (29% and 24% increase, respectively), root length (120% and 77% increase, respectively) and grain protein content (one percentage point increase, from approx. 12% to 13%). The results from this study can help Mediterranean farmers and researchers to develop new fertilization protocols to reach the goals of the “Farm to Fork” European strategy.

## 1. Introduction

The global food demand is expected to increase up to 62% by 2050 [1]. Future agriculture must undoubtedly increase crop production, but it must also do so without increasing the production and use of chemical fertilizers [2]. Indeed, the production and use of nitrogen fertilizer account for approximately 5% of global greenhouse gas (GHG) emissions [3]. However, how to achieve this goal is still uncertain. Because of their potential in alleviating crop stresses and enhancing nutrient use efficiency, plant biostimulants have been proposed as an effective tool to accomplish the so-called “sustainable intensification” of agriculture. For this reason, the biostimulant market has gained increasing value over the last decade [4].

Wheat is a fundamental crop for the world population, providing 20% of the daily intake of protein and calories [5]. While durum wheat [*Triticum turgidum* L. subsp. *durum* (Desf.) Husn.] is a minor cereal in the global scenario, it represents a staple crop for the Mediterranean basin, being the main ingredient of pasta, bread, bulgur and cous cous [6]. Despite being one of the largest durum wheat producers, Italy is experiencing the negative effects of climate change on this crop, mainly through rising temperatures and shifting precipitation patterns [7]. Therefore, the average yield of durum wheat has remained stagnant over the last thirty years, despite the constant genetic improvement [8]. The wide range of stresses that occur during the growing season does not allow new high-productivity cultivars to reach their yield potential. For these reasons, implementing wheat fertilization programs with biostimulants appears to be more a necessity than a possibility in modern agriculture. The term biostimulant refers to a wide range of products derived from different macro categories. Usually, non-microbial biostimulants are classified as chitosan (Chi), humic and fulvic acids (HFA), animal and vegetal protein hydrolysates (PHs), phosphites (Phi), seaweed extracts (SWE), plant extract (PE), and silicon (Si); meanwhile, microbial biostimulants are basically classified as arbuscular mycorrhizal fungi (AMF) and plant growth-promoting rhizobacteria (PGPR) [9,10]. This plethora of products, together with the contrasting results available in the literature, and with variable efficacy due to the mode and time of application, cause great confusion for farmers, who are frequently reluctant to change their conventional fertilization strategy [11,12]. Different biostimulants have different ways to boost plant growth and increase crop performance, such as increasing plant tolerance to abiotic stresses or promoting nutrient uptake and mobilization [13]. Seaweed extracts are reported to enhance root development and stimulate flowering and grain filling in wheat and maize under drought stress, thanks to natural hormones and nutrients [14,15,16,17]. Humic and fulvic acid and amino acids like glycine betaine are reported to increase tolerance to drought and other stresses such as salt and metal pollution [18,19,20]. The plant growth promoting rhizobacteria (PGPR) can produce, in certain conditions, a high amount of phytohormones [21]. Similar effects were reported also for the foliar application of nutrients, such as calcium, an essential element for plants that is involved in the structure of the cell wall and membrane, as well as in intracellular signaling for response to numerous abiotic stresses [22,23]. In a previous study, we tested the efficacy of biostimulant mixtures on durum wheat performance under different levels of nitrogen fertilization [24]. The findings showed a significant effect of foliar application in terms of grain yield and root development, especially at lower nitrogen doses, compared to the untreated control. However, further studies are needed to better evaluate the impact of each single active ingredient. Therefore, a field trial testing five different foliar biostimulants versus the untreated control (conventional fertilization) was conducted with the aim of clarifying the effect of each single formulation on durum wheat performances.

The hypotheses were as follows: (i) all the tested biostimulants significantly enhance the root development and leaf chlorophyll content of durum wheat compared to the conventional fertilization practice; (ii) foliar treatments increase durum yield and quality attributes; (iii) the degree of the biostimulant effect varies with the active ingredients.

## 2. Results

### 2.1. Weather Conditions

**Figure 1:**
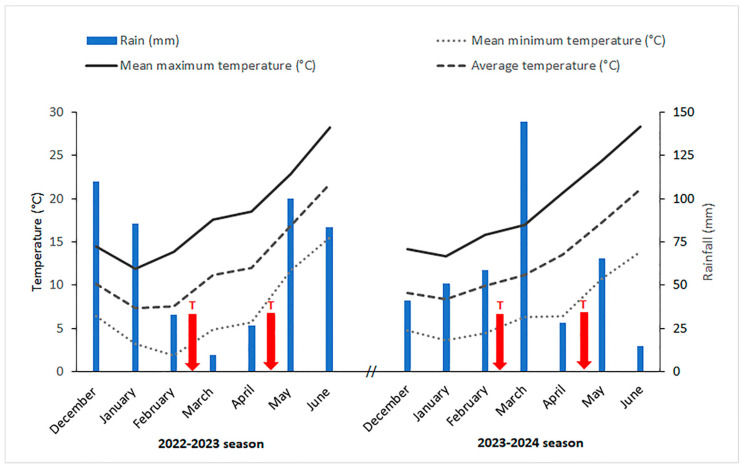
Climatic conditions during the experiment. The biostimulant treatments (T) were applied two times per season, in the points indicated by the arrows.

Seasonal weather patterns showed some differences between the experimental years (Figure 1), especially in terms of total rainfall. Considering the period from sowing to harvest, the 2023 season was characterized by a slightly higher rainfall amount than 2024 (+48 mm). The stem elongation phase of the two growing seasons occurred under completely different weather conditions. In more detail, March 2023 and March 2024 were the months with the lowest and the highest seasonal amount of rain, respectively (less than 10 mm vs. 145 mm). Regarding average and maximum air temperature, the two growing seasons were similar. Conversely, in terms of minimum temperature, February 2023 was colder than February 2024 (approx. 2 °C vs. 4.5 °C).

### 2.2. Crop Traits

As shown in Table 1, biostimulant treatment significantly influenced all the measured traits, except for the number of kernels per spike.

Results of root length, grain yield, quality traits and leaf chlorophyll content are reported in Table 2.

**Figure 2:**
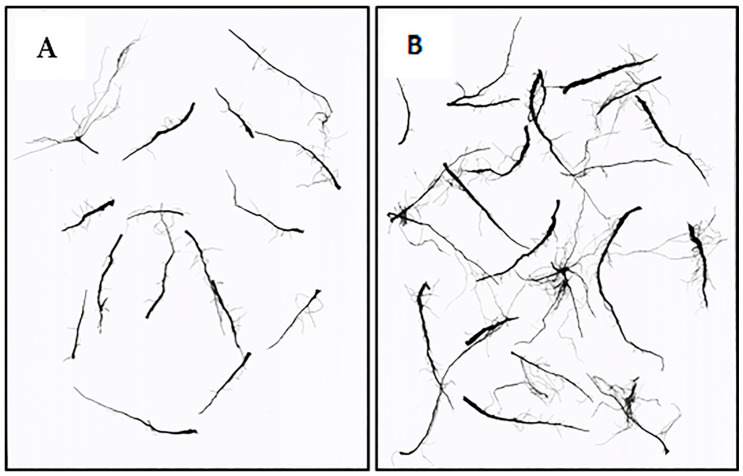
WinRHIZO™ scans of representative durum roots: (**A**) control plant, (**B**) plant treated with T1 (application of extracts of *Codium fragile* and *Opuntia ficus-barbarica*).

The T1 and T0 treatments produced the longest and the shortest roots, respectively (Table 2). Specifically, T1 resulted in a 120% increase in root length as compared to control treatment (385 cm vs. 175 cm, Figure 2). The other biostimulant treatments (except for T4) outperformed T0 with an average root length of 309 cm.

T1 and T2 treatments significantly boosted final grain yield as compared to the control (Table 2). Specifically, T1 increased grain production by 29% (5.3 t ha^−1^ vs. 4.1 t ha^−1^) while T2 by 24% (5.1 t ha^−1^ vs. 4.1 t ha^−1^). The other treatments did not differ significantly from the control.

As reported in Table 2, T1, T2 and T5 treatments enhanced the number of spikes per unit area by 26% as compared to the control (222 vs. 176 spikes m^−2^). The other treatments did not produce a statistically significant effect as compared to T0 treatment.

Regarding the thousand kernel weight, all the biostimulant formulations significantly outperformed the control treatment (Table 2). In more detail, biostimulant application enhanced the seed weight by roughly 10% (54.7 g vs. 49.7 g).

Biostimulant treatment significantly influenced both grain protein content and test weight (Table 1). Only T1 and T2 resulted in a grain protein content significantly higher than the control (Table 2). Specifically, the application of these foliar treatments enhanced the protein concentration by almost one percentage point (from 12.4% to 13.1%).

Concerning test weight, only the T1 treatment significantly outperformed the control (Table 2). Particularly, the application of seaweed extracts was able to increase the test weight by 1%, from 77.6 kg hL^−1^ to 78.5 kg hL^−1^.

**Figure 3:**
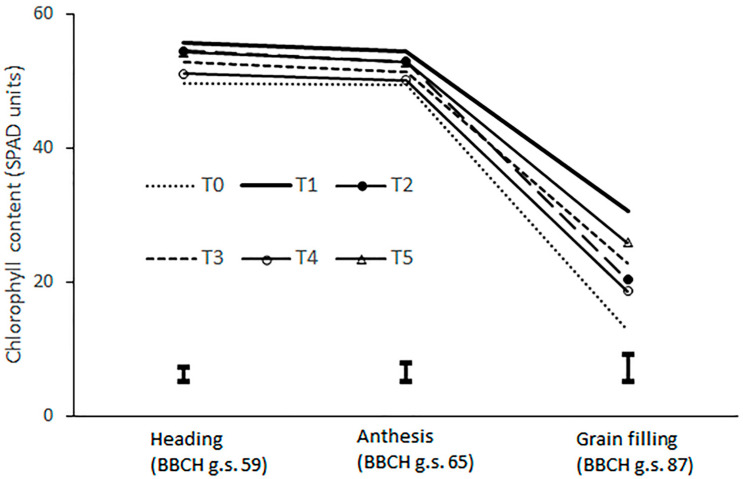
Leaf chlorophyll content of durum wheat as influenced by biostimulant treatment in different growth stages. The vertical bars represent the standard error of the mean. T0: untreated control; T1: application of extracts of *Codium fragile* and *Opuntia ficus-barbarica*; T2: application of micronized vaterite; T3: application of *Pseudomonas protegens*, T4: application of humic and fulvic acid; T5: application of nitrogen fertilizer containing glycine betaine; g.s.: growth stage.

**Figure 4:**
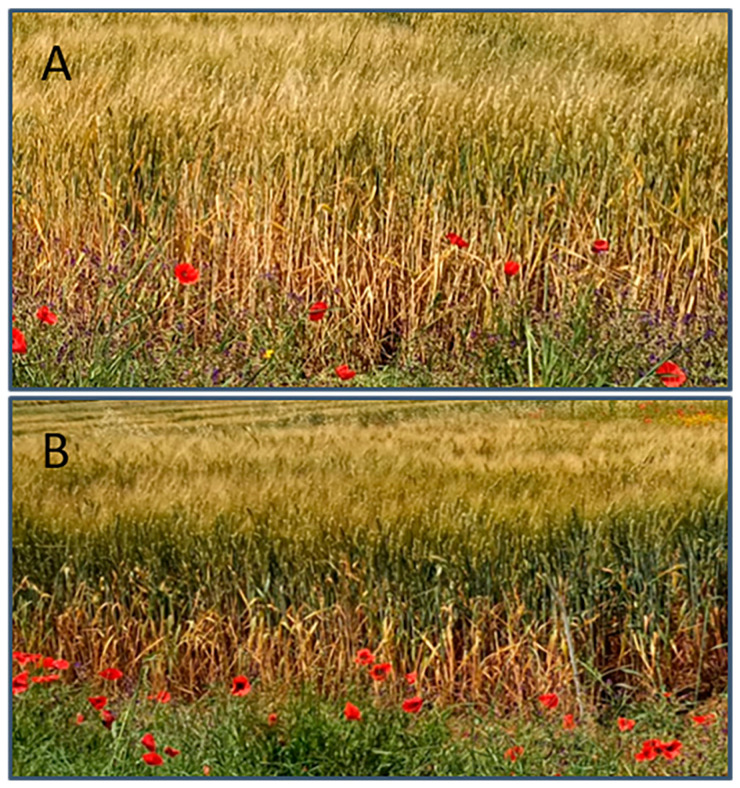
Experimental plots during the grain filling stage. (**A**) Control plants, (**B**) plants treated with T1 (application of extracts of *Codium fragile* and *Opuntia ficus-barbarica*).

Biostimulant application had a significant effect on leaf chlorophyll content in all sampling dates (Table 3). T1, T2 and T5 treatments showed the highest chlorophyll content (10% more than control), with values slightly decreasing from 55 to 53 SPAD units when moving from heading to anthesis (Figure 3). T3 and T4 treatments did not differ statistically from the control. During the grain filling phase, the T1 and T5 treatments showed the highest SPAD values (30.5 and 25.8, respectively), with a 100% to 150% increase as compared to the control (Figure 4). Finally, the T2 and T3 treatments showed a chlorophyll content significantly higher than the control treatment (21.5 vs. 12.7 SPAD units), with an average increase of about 70%.

## 3. Discussion

Increasing food production without increasing input of chemicals is the major task of the modern agriculture in a changing climate [25]. In this study, the potential of each single biostimulant in improving durum wheat yield and quality under the standard nitrogen fertilization rate was explored.

The results from the present field experiment show that the agronomic performance of durum wheat can be boosted with two applications of foliar biostimulants. However, to maximize the response of durum wheat, the proper selection of the active ingredient becomes a crucial point [26].

We found that the foliar treatments containing seaweed and plant extracts (T1) and those with micronized vaterite (T2) significantly increased the grain yield and protein content. These results confirm the positive effect of vaterite on wheat performance [27], further underlining the fundamental role of foliar fertilization in obtaining good yield and grain quality in wheat [28].

As for *Codium fragile* and *Opuntia ficus-barbarica* extracts, these substances were previously found to enhance germination and seedling vigor in controlled environment experiments, under both stress and no stress conditions [29,30,31]. Now, the positive effect was extended to other plant traits, growth stages, and under field conditions. Similar outcomes were reported for other seaweed extracts [32,33], with yield increases that ranged from 5 % to 35% compared to the control.

Conversely, the other treatments containing *Pseudomonas protegens* (T3), humic and fulvic acids (T4) and glycine betaine (T5), did not significantly enhance wheat yield and grain protein content. This was probably due to the favorable climatic conditions which characterized the two growing seasons. Indeed, these substances do have a significant effect on crop production, especially under limiting environmental conditions [34,35,36].

When compared to the control, the significant increase in grain production was mainly driven by the first yield component: the number of spikes per unit area. This effect was also reported in other studies, especially with the foliar applications of extracts from *Spirulina plantensis* and *Ascophyllum nodosum* [37]. The phytohormones contained in the seaweed extracts and in the culture broth of PGPR could be involved in tiller growth and the development of cereals [38,39,40].

Additionally, we found that the T1 treatment doubled the root length of durum wheat as compared to the control. This is a well-known effect of the phytohormones and micronutrients contained in the seaweed extracts, which are fundamental for root development and growth of new tillers [41]. In particular, seaweed extracts from *C. fragile* have repeatedly highlighted this positive effect on durum wheat in germination trials [29,30]. As expected, thanks to the content of phytohormones in the culture broth and the ability of bacteria to synthesize auxins and fix nutrients [42,43], the T3 treatment also led to a significant increase in wheat root development. Finally, the foliar application of glycine betaine also enhanced the root length of durum wheat. This was probably caused by the role that this amino acid plays in activating antioxidant enzymes and drought-responsive hormones [44].

All the tested biostimulants, except for T4, significantly increased leaf chlorophyll concentration, especially in the late growth stages, thus inducing a stay-green phenotype. The best results for this trait were achieved by T1 and T5 treatments, probably because of their content in proline (T1) [45] and glycine betaine (T5). Both amino acids have the capacity to protect the photosynthetic systems of crops [18,46]. Calcium contained in the micronized vaterite also increased the chlorophyll content, thus confirming its role in improving the photosynthesis and related physiological and biochemical attributes of wheat [47]. The foliar application of humic and fulvic acid led to an increase in leaf chlorophyll values, although to a lesser extent than the other biostimulants. These compounds were previously reported to increase the activity of Rubisco, a fundamental enzyme in photosynthesis [48].

Regarding grain quality, the application of biostimulants as foliar treatment during key growth stages could be a valid strategy to enhance grain protein content and test weight while reducing the amount of synthetic nitrogen [24]. In the present study, only T1 treatment significantly increased both the grain protein content and test weight when compared to the control. This result was possibly due to the highest accumulation and translocation of photoassimilates that this treatment achieved during the grain filling phase (stay-green phenotype). However, the effect of seaweed extracts on the quality traits of cereals remains a controversial topic [33,49].

## 4. Materials and Methods

### 4.1. Location and Experimental Design

The field trial was carried out in Viterbo, central Italy (latitude 42°43′ N, longitude 12°07′ E, altitude 310 m), under rainfed conditions, during the growing seasons 2022–2023 and 2023–2024. The weather pattern of the two seasons is reported in Figure 1. A randomized block design with three replicates was used. Individual plots were 6 m^2^ each.

Soil was plowed in summer (30 cm depth) and then harrowed before seeding. Durum wheat cultivar “Iride” was chosen for its excellent environmental adaptability [50] and it was sown on 14 December 2022 in the first season and 26 November 2023 in the second season. Weeds were chemically controlled using a post-emergent herbicide (containing Florasulam and 2,4D) in a single application at the end of tillering.

The field was fertilized before sowing with diammonium phosphate (18% N–46% P) at the rate of 150 kg ha^−1^. Topdressing nitrogen fertilization was distributed using granular urea (46% N) and dividing the total dose (150 kg N ha^−1^) into two equal rates: the first applied at late tillering (BBCH growth stage 25) and the second at flag leaf emergence (BBCH growth stage 37).

Five different biostimulants were tested in comparison with the untreated control (T0):

- (T1), extracts of seaweed *Codium fragile* (Suringar) Hariot and plant *Opuntia ficus-barbarica* A. Berger at the dose of 1 kg ha^−1^. After they were harvested, the algae were thoroughly washed with fresh water and then ground. Mechanical pressure was used to extract the desired compounds from the seaweed biomass. The weight ratio of the algae and plant pressed biomass mixtures was 5:1;
- (T2), micronized vaterite, calcium carbonate (Ca 29%), with particle size lower than 5 µm, at the dose of 2 kg ha^−1^;
- (T3), culture broth of *Pseudomonas protegens* (10^9^ CFU g^−1^, determined by serial dilution method on Petri dish), rich in auxins and cytokinins, at the dose of 1 kg ha^−1^. *Pseudomonas protegens* was fermented at room temperature in a continuously stirred vessel with a substrate containing water, molasses, ammonium sulfate and tryptophan;
- (T4), humic and fulvic acid enriched in micronutrients such as iron and zinc at the dose of 1 kg ha^−1^. The product derived from the extraction of leonardite with KOH. It contained 62% organic matter (DM basis), with 1.1% of organic nitrogen and the following nutrients: P_2_O_5_ (238 ppm), SO_3_ (681 ppm), CaO (939 ppm), Fe (253 ppm), Cu (96 ppm), Mg (78 ppm), Zn (71 ppm), B (71 ppm), Mo (28 ppm) and Mn (25 ppm);
- (T5), organic nitrogen fertilizer (N 5%) derived from sugar-beet processing and containing 35% of glycine betaine, at a dose of 5 kg ha^−1^. The product also contains 15% C and 1.5% K_2_O.

All the biostimulants were dissolved in water and applied by spraying the canopy at the end of tillering (BBCH growth stage 25–26) and at heading (BBCH growth stage 59), as shown in Figure 1. The amount of water distributed in a single treatment was calculated considering the standard dose of 300 kg ha^−1^; the same amount of water was distributed also to the control plots.

### 4.2. Sampling and Measurements

Data on the following plant traits were collected during the study: leaf chlorophyll content, root length, grain yield (13% moisture), number of spikes per unit area, number of kernels spike^−1^, 1000-kernel weight, grain protein content, and test weight.

Chlorophyll content was measured on flag leaf during heading (BBCH growth stage 59), anthesis (BBCH growth stage 65), and late grain filling (BBCH growth stage 87) by using a hand-held meter SPAD 502 (Konica-Minolta Inc., Hino-shi Tokyo, Japan). Five flag leaves per each plot were measured and then averaged to obtain the replicate value.

The root sampling method was described in our previous work [24] and the analysis was performed using WinRHIZO™ (version 2016) scanning and software [51]. Images were scanned at 600 dpi (945 pixels) resolution. Root length was calculated by multiplying the pixel number in the root skeleton by the pixel dimension [52,53]. Three plants for each plot were used for root analysis and measures were averaged to obtain the replicate value.

Grain yield was measured by harvesting the whole plot (excluding the external rows) with a plot harvester and weighing the resulting production. Furthermore, to express the yield at 13% standard moisture, a grain sample was dried to determine the harvest moisture and the following formula was applied:

$$Grain\;yield\;(13\%\;moisture)=\;\frac{\left(Harvest\;yield)\;\times \;(1\;-\;Harvest\;moisture\right)}{\left(1\;-\;Standard\;moisture\right)}$$

where Harvest yield = grain yield at the time of harvest; Harvest moisture = moisture at harvest determined after drying grain samples; Standard moisture = 0.13.

Regarding durum wheat quality (protein content and test weight), grain samples (250 g each) from each plot were ground using a PerkinElmer LM-3610 grinder (PerkinElmer Health Sciences Canada Inc., Winnipeg, MB, Canada) equipped with a 1 mm sieve. Grain protein content (expressed as %) was measured scanning the samples with a benchtop NIR (Near-Infrared Reflectance) analyzer (FOSS-DS2500; FOSS Electric A/S, Hillerød, Denmark). Finally, the test weight was determined by using the Schopper chondrometer, and transforming the obtained values in kg hL^−1^.

### 4.3. Statistical Analysis

R software, version 3.5.2 [54], was used to perform the analysis of variance (ANOVA) for the measured data. Normality and homoscedasticity were verified before running the one-way ANOVA. The year effect was considered as random. Tukey test (*p* < 0.05) was performed as post hoc test.

## 5. Conclusions

While the first two hypotheses of the present study were not always verified (because some biostimulants failed in producing a significant effect on some durum wheat traits), the third hypothesis must be accepted. Indeed, our study clearly showed the differential response of durum wheat when treated with different biostimulant substances. Particularly, the applications of *Codium* and *Opuntia* extracts as well as micronized vaterite were the only two treatments that could really improve both the agronomic performance and grain protein content of durum wheat cultivar “Iride”, as compared to the untreated control.

The results from this study can help Mediterranean farmers and researchers to develop new sustainable fertilization protocols to reach the goals of the “Farm to Fork” European strategy.

To this aim, additional studies are needed to test the potential of these different biostimulants on other durum wheat cultivars as well as other important food crops, under stressful conditions, in broader field trials, and through multi-season experiments.

## Figures and Tables

**Table 1:** ANOVA table for root length, grain yield, yield components and grain quality of durum wheat.

	Biostimulant
Root length	***
Grain yield	**
Number of spikes m^−2^	***
Number of kernels spike^−1^	ns
Thousand kernel weight	**
Grain protein content	***
Test weight	**

Levels of significance: *** < 0.001; ** < 0.01; ns: not significant. Test weight: Hectoliter weight of grains, expressed as kg hL^−1^.

**Table 2:** Root length, grain yield, yield components and grain quality traits of durum wheat as influenced by biostimulant treatment. The values are means ± standard error. For each trait, letters correspond to the ranking of Tukey’s test at *p* < 0.05. T0: untreated control; T1: application of extracts of *Codium fragile* and *Opuntia ficus-barbarica*; T2: application of micronized vaterite; T3: application of *Pseudomonas protegens*, T4: application of humic and fulvic acid; T5: application of organic nitrogen fertilizer containing glycine betaine. NSM: number of spikes per square meter; TKW: thousand kernel weight.

	T0	T1	T2	T3	T4	T5
Root length (cm)	175 ± 52.9 C	385 ± 52.9 A	318 ± 52.9 AB	309 ± 52.9 AB	249 ± 52.9 BC	301 ± 52.9 AB
Grain yield (t ha^−1^)	4.11 ± 0.4 C	5.26 ± 0.4 A	5.08 ± 0.4 AB	4.47 ± 0.4 AC	4.3 ± 0.4 BC	5.08 ± 0.4 AC
NSM (n. m^−2^)	176 ± 14.7 B	221 ± 14.7 A	223 ± 14.7 A	181 ± 14.7 B	181 ± 14.7 B	222 ± 14.7 A
TKW (g)	49.7 ± 1.25 B	55.7 ± 1.25 A	53.2 ± 1.25 A	54.4 ± 1.25 A	54.8 ± 1.25 A	55.3 ± 1.25 A
Protein content (%)	12.4 ± 0.38 C	13.3 ± 0.38 A	12.9 ± 0.38 AB	12.4 ± 0.38 C	12.7 ± 0.38 BC	12.4 ± 0.38 C
Test weight (kg hL^−1^)	77.6 ± 0.91 B	78.5 ± 0.91 A	78.2 ± 0.91 AB	78.2 ± 0.91 AB	78.0 ± 0.91 AB	78.1 ± 0.91 AB

**Table 3:** ANOVA table for leaf chlorophyll content measured in different durum growth stages.

	Heading	Anthesis	Grain Filling
	(BBCH stage 59)	(BBCH stage 65)	(BBCH stage 87)
Biostimulants	***	***	***

Levels of significance: *** < 0.001.

## Data Availability

The data presented in this study are available on request from the corresponding author.
